# The Impact of NOTCH Pathway Alteration on Tumor Microenvironment and Clinical Survival of Immune Checkpoint Inhibitors in NSCLC

**DOI:** 10.3389/fimmu.2021.638763

**Published:** 2021-07-09

**Authors:** Xiaohua Li, Yuntao Wang, Xuebing Li, Gang Feng, Sheng Hu, Yifeng Bai

**Affiliations:** ^1^ Department of Respiratory and Critical Care Medicine, Sixth People's Hospital of Chengdu, Chengdu, China; ^2^ Department of Oncology, The Fifth People’s Hospital Affiliated to Chengdu University of Traditional Chinese Medicine, The Second Clinical Medical College, Chengdu, China; ^3^ Department of Respiratory and Critical Care Medicine, People's Hospital of Yaan, Yaan, China; ^4^ Department of Thoracic Surgery, Sichuan Provincial People’s Hospital, University of Electronic Science and Technology of China, Chengdu, China; ^5^ Department of Respiratory and Intensive Care Medicine, The General Hospital of Western Theatre Command, Chengdu, China; ^6^ Department of Oncology, Sichuan Provincial People’s Hospital, University of Electronic Science and Technology of China, Chengdu, China

**Keywords:** NOTCH signaling, non-small cell lung cancer, immune checkpoint inhibitors, tumor microenvironment, immunogenicity

## Abstract

The treatment of non-small cell lung cancer (NSCLC) with immune checkpoint inhibitors (ICIs) has been proven to induce lasting tumor remission. Screening suitable populations for immunotherapy through predictive markers is an important approach to improving the clinical benefits of patients. Evidence has shown that there may be a close connection between NOTCH signaling and the tumor microenvironment (TME). Hence, we explored the impact of the mutation status of NOTCH signaling on the prognosis of NSCLC patients treated with immunotherapy with the aim to apply NSCLC immunotherapy to the greatest extent possible. We examined two clinical cohorts of NSCLC patients receiving ICIs (MSKCC and NG cohorts). The mutation and prognostic data of the ICI-treated cohort were used to evaluate the association between the mutation status of NOTCH signaling and prognosis following immunotherapy. The expression and mutation data of The Cancer Genome Atlas (TCGA)-NSCLC cohort were used to analyze the differences in the immune microenvironment under different NOTCH signaling mutation states. In the ICI-treated cohorts, the univariate and multivariate Cox regression analyses indicated that high-mutated NOTCH signaling could serve as an independent predictor of NSCLC patients receiving ICIs. Patients with high-mutated NOTCH signaling had significantly improved progression-free survival (PFS) (P = 0.03, HR = 0.69; MSKCC cohort) and prolonged overall survival (OS) (P = 0.004, HR = 0.34; NG cohort). Additionally, high-mutated NOTCH signaling was related to the inflammatory immune microenvironment, inflammatory expression profile, and enhanced immunogenicity. According to this study, high-mutated NOTCH signaling may serve as a biomarker for the prediction of the prognosis of NSCLC patients treated with ICIs. A series of prospective clinical studies and molecular mechanism explorations are still needed in the future.

## Introduction

Currently, lung cancer is a common malignant tumor worldwide, and its incidence is increasing yearly, resulting in approximately 25% of cancer-related deaths (1). Among lung cancer patients, approximately 85% have non-small cell lung cancer (NSCLC), and most patients are at the advanced stage at diagnosis (2). Immune checkpoint inhibitors (ICIs) have achieved many benefits in multiple cancer types. In advanced NSCLC, programmed cell death 1 (PD-1)/programmed cell death ligand 1 (PD-L1) have the most application prospects (3, 4). ICIs can significantly improve the survival of cancer patients, with a 5-year survival rate of approximately 15%-50% (5–8). However, in the unselected population, only a small number of patients can benefit from ICIs (4, 9). Therefore, the identification of suitable markers of efficacy has become an important part of immunotherapy.

Studies have shown that PD-L1 and PD-L2 expression, the tumor mutational burden (TMB), tumor infiltrating lymphocytes (TILs), proinflammatory factors and gene mutations are related to the immune efficacy of NSCLC (9, 10). However, growing evidence has shown that PD-L1 expression, the TMB, and microsatellite instability-high (MSI-H) still cannot accurately screen most patients with NSCLC (11–14). For example, in some studies, no correlation between PD-L1 expression and the response to ICIs or overall survival (OS) was found (15). Possible potential reasons for this result include the different detection methods used, PD-L1 expression times, the use of nonstandardized criteria to assess the positive rate, and cutoffs. Chowell et al. discovered that some patients with a low TMB could also respond to immunotherapy and that patients with a high TMB may not show good immunotherapy efficacy (16). Therefore, screening predictive biomarkers has become an urgent challenge in clinical applications.

Recently, mutations in specific pathways have been used as predictive markers for immunotherapy in many cancer patients (17, 18). It has been confirmed that NOTCH signaling is closely associated with the tumor microenvironment (TME) as it regulates tumor angiogenesis and immune cell infiltration and maintains tumor cell stemness. For example, NOTCH signaling plays an important role in activating cytotoxic T cells (CTLs), promoting the maturation of naive CD8+ T cells and expressing granzyme B and IFN-γ (19). Additionally, NOTCH signaling can increase the phenotype of proinflammatory macrophages (M1), and the antigen presentation activity of macrophages lacking NOTCH signaling is reduced (20). Moreover, NOTCH signaling mediates the secretion of many molecules (such as IL-6, IL-10, TGF-β, CCL2 and CXCL12) and affects the functions of cells in the TME (21–23). Wang et al. found that mutations in the NOTCH signaling pathway are associated with a satisfactory immunotherapy prognosis in patients with colorectal cancer (CRC). Additionally, mutations in the NOTCH signaling pathway are associated with high expression levels of PDCD1, CTLA4 and CD274 in CRC (24). Hence, we aimed to explore the impact of the mutation status of NOTCH signaling on the prognosis of NSCLC patients treated with ICIs to ultimately better apply NSCLC immunotherapy to the greatest extent possible.

## Methods

### Clinical Samples

We downloaded data from an ICI-treated cohort (25) including 240 NSCLC patients treated with ICIs from cBioPortal to explore the relationship between the mutation status of NOTCH signaling and the prognosis of NSCLC patients receiving ICIs. Another ICI-treated cohort (Miao et al.) was used as a validation set in this analysis (26). The RNA sequencing and whole-exome sequencing (WES) data of The Cancer Genome Atlas-Lung Adenocarcinoma (TCGA-LUAD) and TCGA-Lung Squamous Cell Carcinoma (LUSC) cohorts were collected from the TCGA database (27). We collected 35 formalin-fixed paraffin-embedded (FFPE) NSCLC samples from the Sichuan Provincial People’s Hospital, University of Electronic Science and Technology of China and performed panel-sequencing. The NSCLC tumor samples, data sequencing and processing are described in detail in the Supplementary Methods.

### Data Preprocessing

Using the mutation data, we a conducted follow-up analysis of only the nonsynonymous mutation data. The NOTCH signaling gene set was derived from the Molecular Signatures Database (MSigDB) (28). In each sample, we calculated the number of nonsynonymous mutations in NOTCH signaling. According to the median number of nonsynonymous mutations in the NOTCH signaling in each cohort, the patients were classified according to their NOTCH signaling mutation status and divided into the high-mutated and low-mutated NOTCH groups. After defining the sample groupings, we estimated the tumor mutational burden (TMB) of each patient based on the reported literature (29). We also obtained information regarding the neoantigen load (NAL) and immune scores from the published literature (30). The gene sets related to DNA damage repair were obtained from the MsigDB (28). We also evaluated the number of nonsynonymous mutations in the DNA damage response (DDR) pathway.

### Immune Infiltration Algorithm

The CIBERSORT algorithm was applied to evaluate the abundance of 22 immune cell types in the TME of NSCLC (31). The genes related to immune checkpoints and immunity were derived from previous research reports (30). A gene set enrichment analysis (GSEA) was used to analyze and compare the differences in the activities of pathophysiological pathways, such as those derived from Gene Ontology (GO), Kyoto Encyclopedia of Genes and Genomes (KEGG) and REACTOME, between the high-mutated and low-mutated NOTCH groups (32).

### Statistical Analysis

Univariate and multivariate Cox regression models were used to analyze the effect of the high- and low-mutated NOTCH statuses on the clinical prognosis of NSCLC patients treated with ICIs. The hazard ratio (HR) and 95% confidence interval (CI) were used to evaluate the models. We used a Mann-Whitney U test to compare the differences in the continuous variables between the high-mutated NOTCH group and low-mutated NOTCH group. We used Fisher’s exact test to compare the differences in the categorical variables between the high-mutated NOTCH group and low-mutated NOTCH group. Kaplan-Meier (KM) survival curves were drawn to evaluate the relationships between the NOTCH signaling mutation status and the OS and progression-free survival (PFS) of NSCLC patients receiving ICIs, and the log-rank P-value was used to evaluate the significant differences. In this study, a P-value less than 0.05 was considered statistically significant, and all analyses were performed in R software.

## Results

### High-Mutated NOTCH Signaling Can Be Used as an Independent Predictor of the Prognosis of Immunotherapy in NSCLC Patients

**Figure 1:**
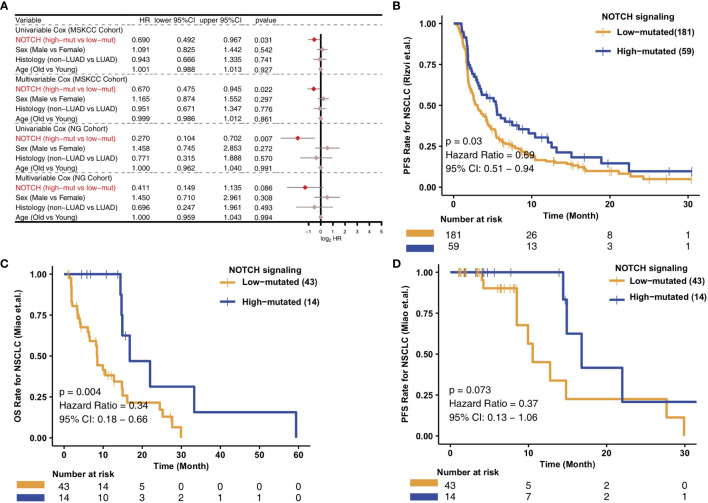
Predictive value of clinical characteristics and the NOTCH signaling mutation status on ICI efficacy. **(A)** Forest plot of the results of the univariate and multivariate Cox regression analyses of the MSKCC and NG cohorts. The main portion of the forest plot presents the hazard ratio (HR) and 95% confidence interval (95% CI), where the red dots indicate p < 0.05. The HR indicates the predictors of favorable (HR < 1) or poor (HR > 1) outcomes. **(B)** KM survival curves of PFS in 240 NSCLC patients from the MSKCC cohort. **(C)** KM survival curves of OS in 57 NSCLC patients from the NG cohort. **(D)** KM survival curves of PFS in 57 NSCLC patients from the NG cohort.

To analyze the relationships between the high- and low-mutated NOTCH statuses and the clinical prognosis of NSCLC patients receiving ICIs, we constructed univariate and multivariate Cox regression models and included other clinical characteristic variables. In the MSKCC cohort, we found that common clinical characteristics, such as sex, histological classification and age, were not related to the prognosis of NSCLC patients after immunotherapy. However, high-mutated NOTCH signaling was related to a good prognosis of immunotherapy in NSCLC patients and could be used as an independent prognostic factor of immunotherapy (**Figure 1A**). To explore the versatility of this marker, we investigated another cohort of NSCLC patients receiving immunotherapy. Similarly, univariate and multivariate Cox regression models were applied to this validation set. The common clinical features could not predict the prognosis of NSCLC patients receiving ICIs, and high-mutated NOTCH signaling could be used as an independent predictor of immunotherapy in NSCLC patients in the NG cohort (**Figure 1A**). In the MSKCC cohort, the KM survival curve showed that the patients with high numbers of mutations in NOTCH signaling (n ​= 59) had significantly better PFS than the patients with low numbers of mutations in NOTCH signaling (P = 0.03, HR = 0.69, 95% CI: 0.51-0.94; **Figure 1B**). In the NG cohort, the KM survival curve showed that the patients with high numbers of mutations in NOTCH signaling (n ​= 14) had significantly prolonged OS (P = 0.004, HR = 0.34, 95% CI: 0.18-0.66; **Figure 1C**). However, we did not observe a significant association between the NOTCH mutation status and PFS in the NG cohort (**Figure 1D**). The univariable Cox analyses of these hallmark pathways related to well-defined biological states or processes showed no significance in the univariable-cox analyses (**Figure S1**).

### Genetic Panorama and Clinical Characteristics of NSCLC Patients With Different NOTCH Signaling Mutation Statuses

**Figure 2:**
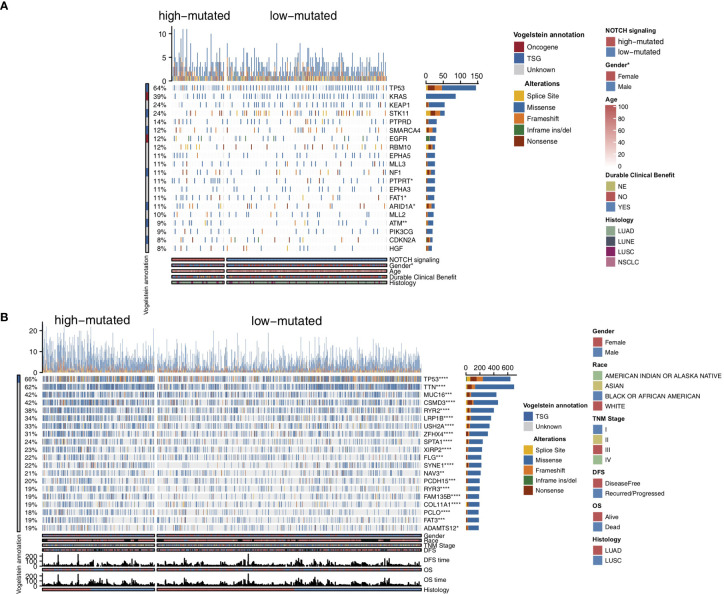
Genomic profiles of the NSCLC patients in the MSKCC **(A)** and TCGA **(B)** cohorts. The top 20 genes with the highest mutation frequencies and the corresponding clinical information are shown in the figure. The top five genes with the highest mutation frequencies in the MSKCC cohort were TP53, KRAS, KEAP1, STK11 and PTPRD. The top five genes with the highest mutation frequencies in the TCGA cohort were TP53, TTN, MUC16, CSMD3 and RYR2. The mutation types are indicated as follows: yellow indicates splice sites, blue indicates missense mutations, orange indicates frameshift mutations, green indicates inframe insertions/deletions, and brown indicates nonsense mutations. The clinical characteristics are shown as patient annotations. (*P < 0.05; **P < 0.01; ***P < 0.001; ****P < 0.0001; Mann-Whitney U test).

**Figure 3:**
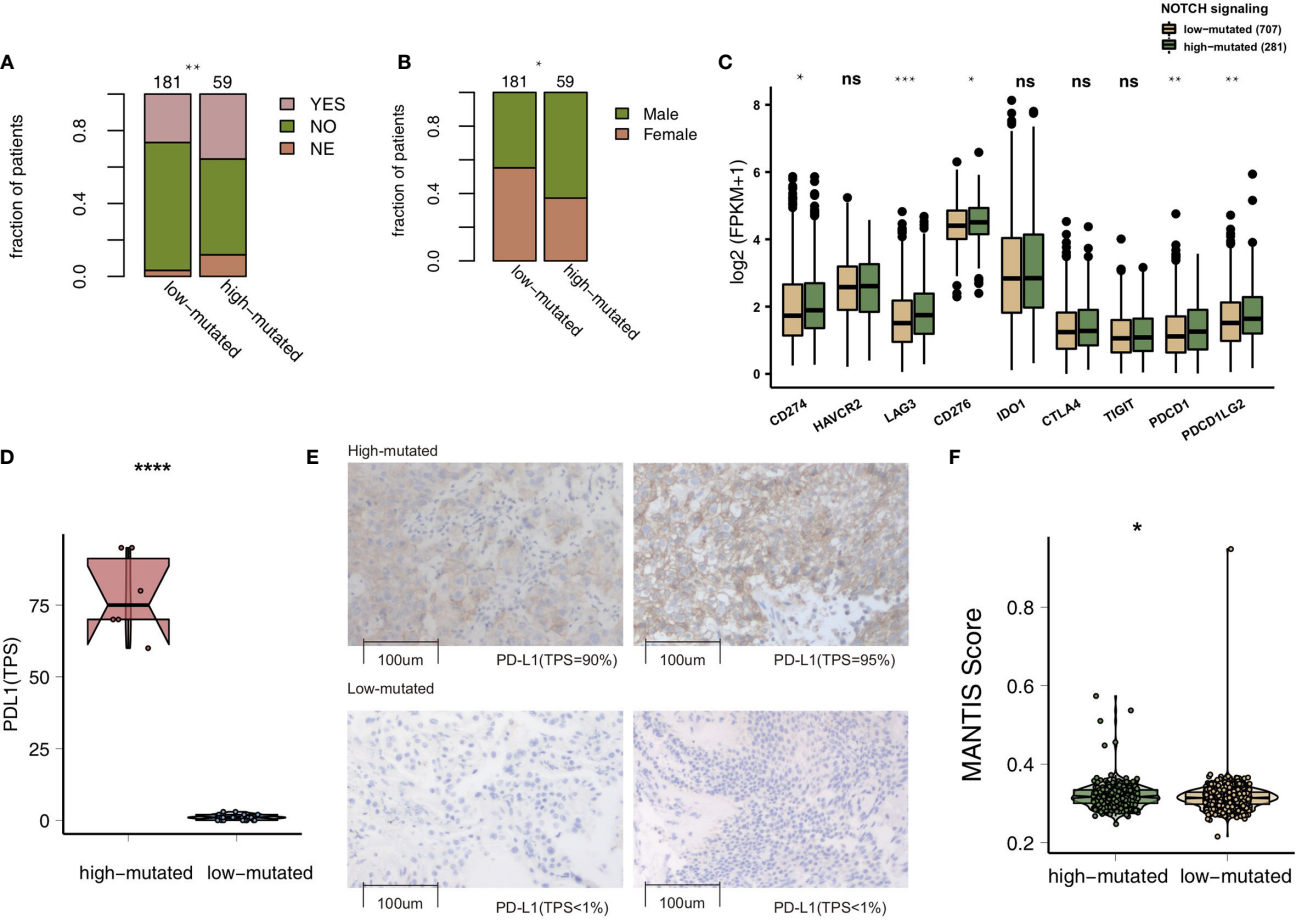
**(A)**. Bar plot showing the proportion of patients in the MSKCC cohort who experienced durable clinical benefits. **(B)** Bar plot showing the proportion of patients in the MSKCC cohort according to sex. The range of p-values is presented by the asterisks above the bar plot (*P < 0.05; **P < 0.01; ***P < 0.001; ****P < 0.0001; Fisher’s exact test). **(C)** Box plots comparing the expression levels of immune checkpoint molecules between the high-mutated and low-mutated NOTCH groups. The range of p-values is presented by the asterisks above each box plot (*P < 0.05; **P < 0.01; ***P < 0.001; ****P < 0.0001; Mann-Whitney U test). **(D)** Box plots comparing the expression levels of PD-L1 (TPS) between the high-mutated and low-mutated NOTCH groups in the Local cohort (****P < 0.0001; Mann-Whitney U test). **(E)** Typical cases of the PD-L1 (TPS) level in the high- (2 samples) and low-mutated (2 samples) groups in the Local cohort. **(F)**. Violin plots showing the MANTIS score. The range of p-values is presented by the asterisks above the violin plot (*P < 0.05; **P < 0.01; ***P < 0.001; ****P < 0.0001; Mann-Whitney U test). TPS, tumor proportion score; NSCLC, non-small cell lung cancer; TCGA, The Cancer Genome Atlas; PD-L1, programmed cell death-ligand 1. NS, not significant.

To compare the genomic differences in NSCLC patients with different NOTCH mutation statuses, we visualized and compared the mutation data between the MSKCC and TCGA cohorts. Among the top 20 mutated genes in the MSKCC cohort, only some genes (e.g., PTPRT (18.6% *vs* 7.7%, P < 0.05), FAT1 (16.9% *vs* 7.7%, P < 0.05), ARID1A (16.9% *vs* 7.2%, P < 0.05) and ATM (16.9% *vs* 5.0%, P < 0.05); **Figure 2A**) exhibited higher mutation frequencies in the high-mutated NOTCH group than in the low-mutated NOTCH group. Regarding most oncogenes and tumor suppressor genes (TSGs), there was no significant difference between the high-mutated and low-mutated NOTCH groups likely because only targeted sequencing was used in the MSKCC cohort. In the TCGA-NSCLC cohort, the high-mutated NOTCH group had a significantly higher mutation frequency in each of the top 20 mutated genes than the low-mutated NOTCH group (all P < 0.05; **Figure 2B**). The mutual exclusivity and cooccurrence analyses of the top 20 mutated genes are shown in **Figure S2**. The clinical characteristics, such as sex, race, stage and pack years, did not significantly differ between the high-mutated and low-mutated NOTCH groups. In the MSKCC cohort, we found that the efficacy of immunotherapy was higher in the high-mutated NOTCH group, and a larger proportion of patients in this group experienced durable clinical benefits (P < 0.01; **Figure 3A**). Regarding sex, the high-mutated NOTCH group had a higher proportion of males (P < 0.05; **Figure 3B**). The expression levels of important targets of ICIs, such as PD1 (PDCD1), PD-L1 (CD274), LAG3, B7-H3 (CD276), and PDCD1LG2, in the high-mutated NOTCH group were significantly higher than those in the low-mutated NOTCH group (**Figure 3C**). In the Local cohort, the level of PD-L1 in the high-mutated group was significantly higher than that in the low-mutated NOTCH group (**Figure 3D**, P < 0.05). Moreover, the typical IHC of the high-mutated and low-mutated groups is shown in **Figure 3E**. The MANTIS score was used as a signature to evaluate the MSI status. Studies have shown that the MANTIS score is related to the MSI-H status. In the TCGA-NSCLC cohort, the high-mutated NOTCH group had a significantly higher MANTIS score than the low-mutated NOTCH group (P < 0.05; **Figure 3F**).

### High-Mutated NOTCH Signaling Is Associated With Enhanced Immunogenicity

**Figure 4:**
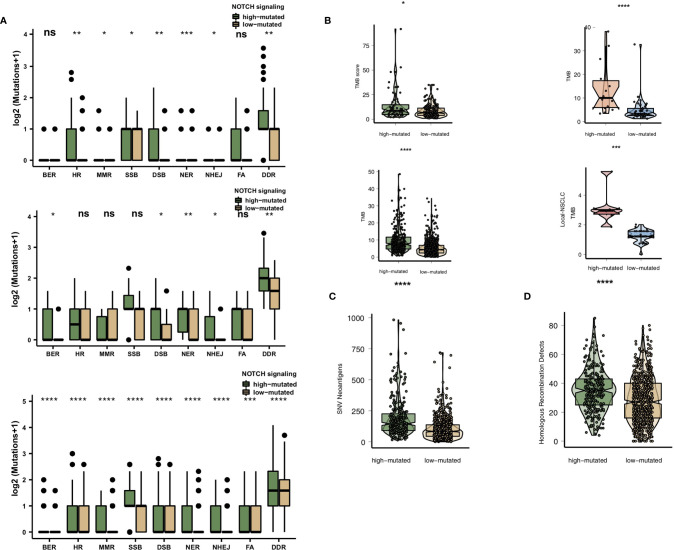
**(A)**. Comparison of DNA damage-related gene set alterations between the high-mutated and low-mutated NOTCH groups in the MSKCC, NG and TCGA-NSCLC cohorts. **(B)** Comparison of the TMB between the high-mutated and low-mutated NOTCH groups in the MSKCC, NG, TCGA-NSCLC and Local cohorts. **(C)** Comparison of NAL between the high-mutated and low-mutated NOTCH groups in the TCGA-NSCLC cohort. **(D)** Comparison of the number of homologous recombination defects between the high-mutated and low-mutated NOTCH groups in the TCGA-NSCLC cohort. NSCLC, non-small cell lung cancer; TCGA, The Cancer Genome Atlas; TMB, tumor mutational burden; NAL, neoantigen load; FA, Fanconi anemia; HR, homologous recombination; NHEJ, nonhomologous end joining; BER, base excision repair; MMR, mismatch repair; NER, nucleotide excision repair; DSB, double-strand break; SSB, single-strand break. (*P < 0.05; **P < 0.01; ***P < 0.001; ****P < 0.0001; Mann-Whitney U test). NS, not significant.

High immunogenicity is easily recognized by TILs in the TME. First, we compared the differences in the numbers of mutated genes in the DDR pathway between the high-mutated and low-mutated NOTCH groups. Regardless of whether the patients received immunotherapy (MSKCC cohort and/or NG cohort) or traditional therapy (TCGA cohort), the number of mutated DDR genes in the high-mutated NOTCH group was significantly higher than that in the low-mutated NOTCH group (**Figure 4A**). In the MSKCC, NG, TCGA and Local cohorts, the high-mutated NOTCH group had a higher TMB than the low-mutated NOTCH group (all P < 0.05; **Figure 4B**).

We also found that the NAL and homologous recombination defect scores in the high-mutated NOTCH group were significantly higher than those in the low-mutated NOTCH group (P < 0.05; **Figures 4C, D**).

### High-Mutated NOTCH Signaling Is Associated With an Inflammatory TME

**Figure 5:**
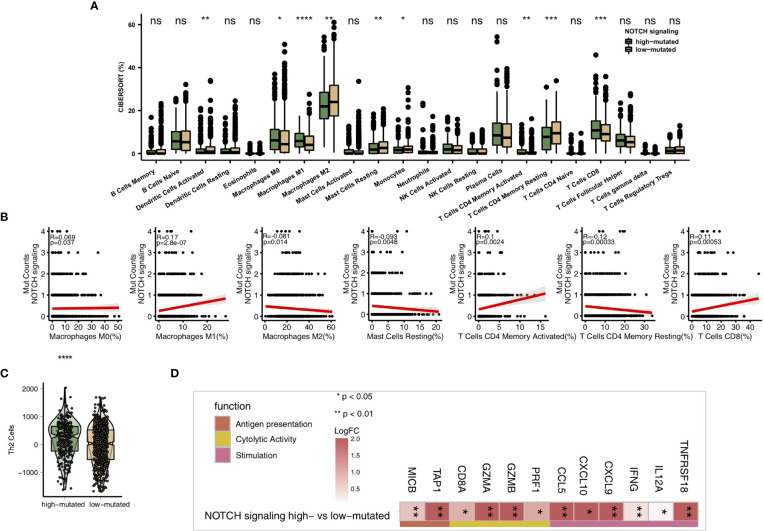
**(A)** Comparison of the proportions of immune cells estimated by the CIBERSORT method between the high-mutated and low-mutated NOTCH groups in the TCGA-NSCLC cohort. **(B)** Correlations between the number of NOTCH signaling mutations and the proportions of each immune cell type. **(C)** Comparison of the Th2 signature between the high-mutated and low-mutated NOTCH groups in the TCGA-NSCLC cohort. **(D)** Comparison of the expression levels of immune-related genes between the high-mutated and low-mutated NOTCH groups in the TCGA-NSCLC cohort. (*P < 0.05; **P < 0.01; ***P < 0.001; ****P < 0.0001; Mann-Whitney U test). NS, not significant.

**Figure 6:**
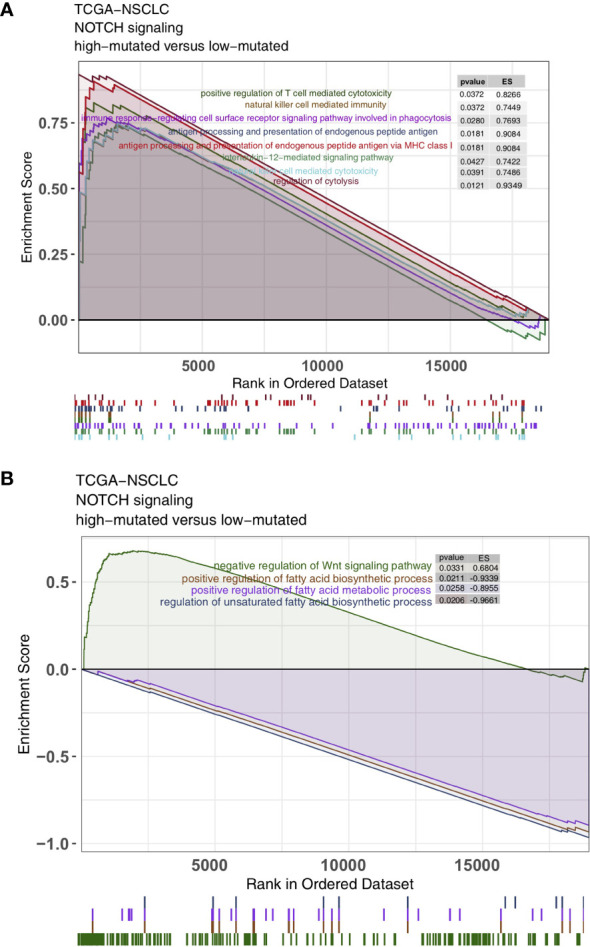
Results of the gene set enrichment analysis (GSEA). **(A)** The immune cell pathways were significantly enriched in the high-mutated NOTCH group. **(B)** The exhausted immunological pathways were significantly downregulated in the high-mutated NOTCH group. The following subgroups served as control groups: low-mutated NOTCH. Enrichment score (ES) > 0 indicates that the corresponding pathways were significantly enriched in the experimental groups. The colors of the curves correspond to the font colors of the pathway names.

As some of the most important components of the TME, TILs play a vital role in ICI treatment. The CIBERSORT algorithm was applied to estimate the abundance of immune cells in NSCLC. In the TCGA-NSCLC cohort, the proportions of various activated immune cells, such as M0 macrophages, M1 macrophages, activated memory CD4+ T cells and CD8+ T cells, in the high-mutated NOTCH group were significantly higher than those in the low-mutated NOTCH group (all P < 0.05; **Figure 5A**). However, the contents of some resting/suppressive immune cells, such as M2 macrophages, quiescent mast cells and resting memory CD4+ T cells, in the low-mutated NOTCH group were significantly higher than those in the high-mutated NOTCH group. Moreover, **Figure 5B** shows that the proportions of activated immune cells, such as M0 macrophages, M1 macrophages, activated memory CD4+ T cells and CD8+ T cells, were significantly positively correlated with the number of mutations in NOTCH signaling (R > 0; P < 0.05). In contrast, a higher proportion of M2 macrophages and/or quiescent mast cells was associated with a lower number of NOTCH signaling mutations (R < 0, P < 0.05; **Figure 5B**). Additionally, we found that the expression levels of some immune-related genes, such as antigen processing and presentation-related genes, cytotoxicity (CYT)-related genes, and inflammatory-related genes, in the high-mutated NOTCH group were significantly higher than those in the low-mutated NOTCH group (log fold change > 0; P < 0.05, **Figures 5C, D**). A GSEA was used to evaluate the pathways that were significantly enriched or downregulated in the high-mutated NOTCH group. The activities of immune-related pathways, such as positive regulation of T cell-mediated cytotoxicity, natural killer (NK) cell-mediated immune response, antigen processing and presentation, and IL-12 pathways, were significantly enriched in the high-mutated NOTCH group (ES > 0, P < 0.05; **Figure 6A**). In contrast, some immune depletion-related signaling pathways, such as fatty acid metabolism-related pathways, were significantly enriched in the low-mutated NOTCH group (ES < 0, P < 0.05; **Figure 6B**).

## Discussion

The use of ICIs for the treatment of NSCLC has been proven to induce long-lasting tumor remission. ICIs can significantly improve the survival status of some NSCLC patients. However, in the unselected treatment population, only a small number of patients can benefit from ICIs. In this study, we found that the NSCLC patients in the high-mutated NOTCH group had a better immunotherapy prognosis than those in the low-mutated NOTCH group. The TME plays a key role in immunotherapy; thus, we explored the potential mechanism by which high-mutated NOTCH signaling improved immunotherapy efficacy from the perspective of the TME. We found that high-mutated NOTCH signaling was associated with significantly increased immunogenicity, activated TILs, upregulated immune checkpoint molecules, and proinflammatory factors. The above results suggest that a high-mutated NOTCH signaling pathway mutation status may be a potential predictive marker for NSCLC patients receiving ICIs.

The inflammatory TME in patients with high-mutated NOTCH signaling may a reason for the better prognosis after receiving ICIs (10, 33, 34). TILs, especially CD4+ and CD8+ T cells and their immunoregulatory cytokines, play a role in adaptive immunity. CD8+ T cells produce IFN-γ, TNF and granzyme B by binding T cell receptors to target tumor cells, leading to tumor cell clearance (17). Studies have suggested that a favorable immunotherapy prognosis in multiple cancer types is related to a high proportion of TILs (35–37). Additionally, chemokines, such as CXCL9/CXCL10, can recruit more CD8+ T cells and NK cells and further mediate the antitumor effects of TILs (38). IFN-γ can not only recruit immune cells to initiate antitumor proliferation and cause tumor apoptosis but also mediate CD8+ T cells to promote ferroptosis in tumor cells (39). NOTCH signaling is critical for the regulation of CTL activation. The activation of naive CD8+ cells requires the combination of Dll1 and NOTCH 1 or NOTCH 2 to express granzyme B and IFN-γ (19). Tumor-associated macrophages (TAMs) can be divided into classically activated macrophages (M1 type) and alternatively activated macrophages (M2 type) according to their cell phenotype. M1 type macrophages mainly secrete and release proinflammatory cytokines, effector molecules and chemokines to kill bacteria, exert antitumor effects, and activate the immune response; M2 type macrophages secrete IL-4/10 and other anti-inflammatory response factors, mediate the secretion of vascular endothelial growth factor (VEGF) and matrix metalloproteinases (MMPs), promote angiogenesis, lymphangiogenesis, tissue reconstruction and damage repair, and suppress immune responses through Th2 cell responses, contributing to the development and metastasis of tumors (10). NOTCH signaling increases the phenotypic polarization of proinflammatory macrophages (M1) (20).

In addition to the immune microenvironment, high inflammation-related expression profiles and high immunogenicity in patients with high-mutated NOTCH signaling may also become potential bases for immunotherapy (34, 40, 41). Jiang et al. used the weighted expression of CD8A, CD8B, GZMA, GZMB, and PRF1 to evaluate the levels of CYT. Moreover, these authors found that a higher CYT score was associated with a better immunotherapy prognosis (40). Growing evidence has confirmed the relationship between tumor immunogenicity, such as the TMB, and the efficacy of ICIs (42–44). It is generally believed that tumors with more mutations may produce more new epitopes that can be recognized by TILs. Studies have also shown that a high NAL is related to the long-term clinical benefits of ICIs in NSCLC patients (44). Mutations in DDR pathway genes play an important role in mediating genomic variation, heterogeneity and instability in tumors (45). Gene mutations in the DDR pathway weaken the ability to repair DNA damage, leading to the accumulation of DNA damage, and are related to the efficacy of PD-1/PD-L1 inhibitors (17, 18). Tumor immunogenicity is also affected by components of the TME, such as professional antigen-presenting cells (pAPCs) (46). MHC-II molecules are expressed mainly by pAPCs, such as dendritic cells (DCs), B cells and macrophages, and present mainly foreign-derived peptide antigens to CD4+ T cells. The expression of tumor-specific MHC-II molecules is related to a good response to ICI treatment (47). As among the most important molecules on the surface of APCs, TAP1 and MICB also play important roles in antigen presentation and processing (30). Consistent with previous studies (48, 49), we also found that patients with highly mutant NOTCH signaling have high expression levels of immune checkpoint molecules, such as PD1 (PDCD1), PD-L1 (CD274), LAG3, B7-H3 (CD276) and PDCD1LG2. Studies have indicated that MHC is associated with the anti-tumor ability of ICIs and may serve as a biomarker of the response to immunotherapy (16, 50, 51). Goodman et al. evaluated the ability of MHC to present NALs using the Patient Harmonic-mean Best Rank (PHBR) scores and found that the poor presentation of NALs by MHC may explain why some tumors did not respond to ICB and were associated with a low TMB (52). In our study, we found that high-mutated NOTCH signaling was associated with a high expression of MHC-related genes. We hypothesized that when high-mutated NOTCH signaling was accompanied by the more efficient presentation of NALs, there was an opportunity for MHC to present NALs, which was critical for the ICIs’ efficacy and response. Additional studies are required to better understand the ability of the combination of NOTCH signaling and the MHC status in the prediction of the response to ICIs among NSCLC patients. Therefore, high immunogenicity (a high TMB, a high NAL, numerous DDR pathway mutations and high levels of antigen presentation-related gene expression) may explain the satisfactory prognosis of patients with high-mutated NOTCH signaling after receiving ICIs.

This study analyzed the prognosis of NSCLC patients treated with ICIs and the mutation status of NOTCH signaling to determine whether high-mutated NOTCH signaling is a possible mechanism for screening the predominant population of NSCLC patients receiving ICIs. However, there are still some limitations. First, targeted sequencing (MSK-IMPACT) was used in the MSKCC cohort to detect somatic mutations, and targeted sequencing provides fewer gene mutations than WES; second, the ICI-treated cohort lacked transcriptomics, copy number variation (CNV), and proteomics data; therefore, the association between high-mutated NOTCH signaling and the prognosis of NSCLC patients treated with ICIs could not be further explored; third, in future research, molecular and animal experiments are needed to further verify our results. Therefore, more studies involving larger samples and diverse ethnic groups are still needed for subsequent analysis and verification.

## Conclusion

According to this study, high-mutated NOTCH signaling may serve as a biomarker for the prediction of the prognosis of NSCLC patients treated with ICIs. In addition to the improved clinical prognosis after ICIs, the patients in the high-mutated NOTCH group had higher immunogenicity, higher proportions of activated immune cells and proinflammatory factors and a higher molecular expression profile related to antigen presentation than the patients in the low-mutated NOTCH group. A series of prospective clinical studies and molecular mechanism explorations are still needed in the future.

## Data Availability Statement

The original contributions presented in the study are included in the article/**Supplementary Material**. Further inquiries can be directed to the corresponding authors.

## Ethics Statement 

Ethical review and approval was not required for the study on human participants in accordance with the local legislation and institutional requirements. Written informed consent for participation was not required for this study in accordance with the national legislation and the institutional requirements. Written informed consent was not obtained from the individual(s) for the publication of any potentially identifiable images or data included in this article.

## Author Contributions

Conceptualization, GF, SH and YFB; Formal analysis, YTW, XHL and XBL; Visualization, YTW, XHL and XBL; Writing - original draft, YTW, XHL and XBL; Writing - review & editing, YTW, XHL, XBL, GF, SH and YFB. All authors contributed to the article and approved the submitted version.

## Funding

This work was supported by grants from the Key research and development project of science and technology department of Sichuan province (2021YFS0128).

## Conflict of Interest

The authors declare that the research was conducted in the absence of any commercial or financial relationships that could be construed as a potential conflict of interest.

## Supplementary Material

Supplementary Figure 1 The univariable Cox analysis including hallmark gene sets from MsigDB.

Supplementary Figure 2 Results of the cooccurrence/mutual exclusivity analysis of the top 20 mutated genes in the MSKCC **(A)** and TCGA-NSCLC **(B)** cohorts.
