# Supplementary figures and images for: The Impact of NOTCH Pathway Alteration on Tumor Microenvironment and Clinical Survival of Immune Checkpoint Inhibitors in NSCLC
Source: Front Immunol. 2021 Jul 9;12:638763. doi: 10.3389/fimmu.2021.638763 (PMC8302260; doi:10.3389/fimmu.2021.638763)

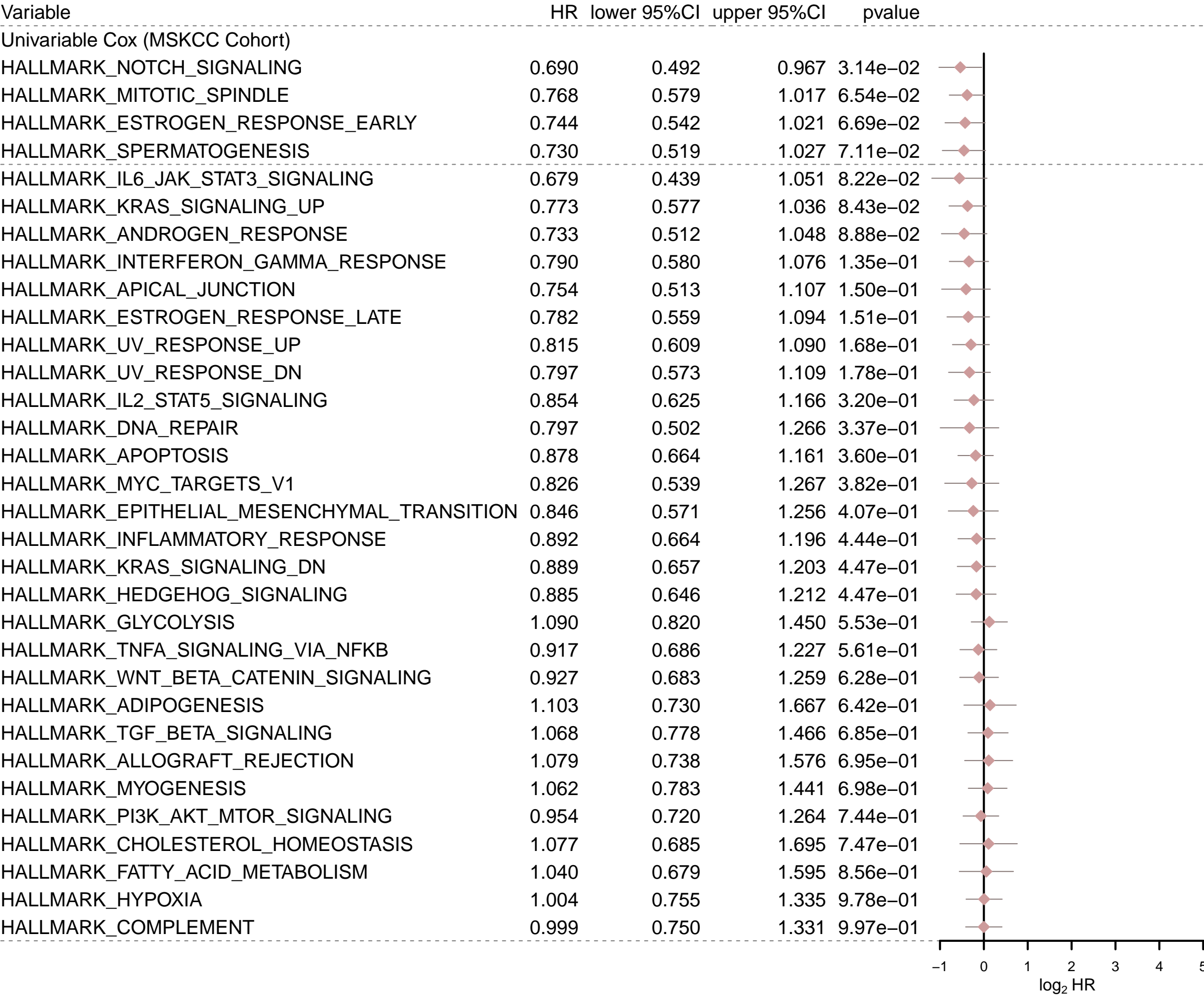

Supplement: Supplementary Figure 1 — The univariable Cox analysis including hallmark gene sets from MsigDB. [file Image_1.pdf]

**A**

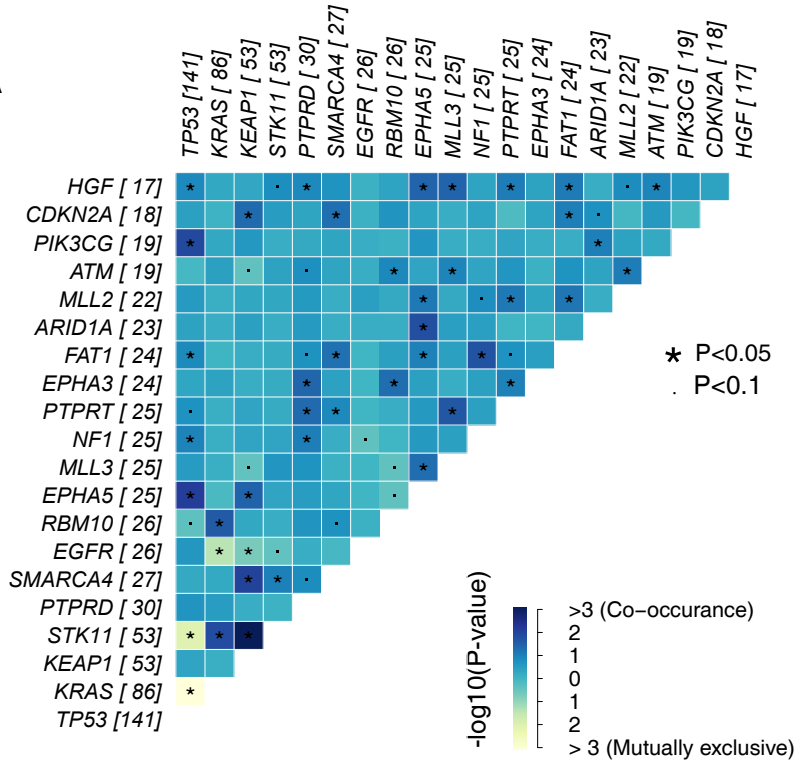

**B**

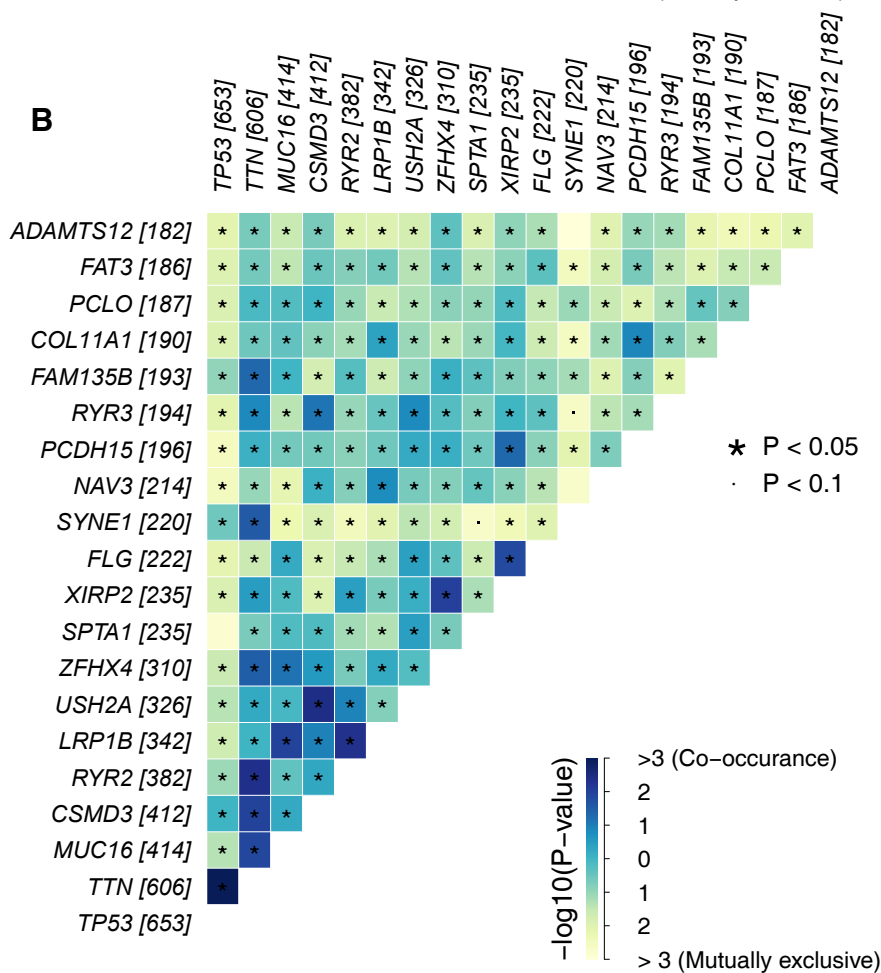

Supplement: Supplementary Figure 2 — Results of the cooccurrence/mutual exclusivity analysis of the top 20 mutated genes in the MSKCC (A) and TCGA-NSCLC (B) cohorts. [file Image_2.pdf]
